# A Comparative Transcriptome Analysis Reveals Physiological Maturation Properties of Mycelia in *Pleurotus tuoliensis*

**DOI:** 10.3390/genes10090703

**Published:** 2019-09-11

**Authors:** Fang Du, Nu Er zi ya Ya Li mai mai ti, Qingxiu Hu, Yajie Zou, Dou Ye, Haijun Zhang

**Affiliations:** 1 Institute of Agricultural Resources and Regional Planning, Chinese Academy of Agricultural Sciences, Beijing 100081, China; 2 Institute of Plant Protection, Xinjiang Academy of Agricultural Sciences/Key Laboratory of Integrated Pest Management on Crop in Northwestern Oasis, Urumqi 830091, China

**Keywords:** *Pleurotus tuoliensis*, mycelium physiological maturation, comparative transcriptomic analysis, nucleoside diphosphate kinase

## Abstract

*Pleurotus tuoliensis* is a precious edible fungus with extremely high nutritive and medicinal value. The cultivation period of *P. tuoliensis* is longer than those of other *Pleurotus* species, which is mainly due to a longer mycelium physiological maturation period (30–60 days). Currently, the molecular processes underlying physiological maturation of the mycelium remain unclear. We performed a comparative transcriptomic analysis of immature and mature mycelia using RNA-seq. De novo transcriptome assembly resulted in identification of 17,030 unigenes. 451 differentially expressed genes—including those encoding nucleoside diphosphate kinase (NDPK), glycoside hydrolase family proteins, exopolygalacturonase, and versatile peroxidases—were identified. Gene Ontology (GO) and Kyoto Encyclopedia of Genes and Genomes (KEGG) analyses revealed that nucleotide synthesis and energy metabolism are highly active during the physiological maturation of mycelia, and genes related to these pathways were significantly upregulated in mature mycelia. NDPK is predicted to be essential for mycelia maturation. Our findings contribute to a comprehensive understanding of mycelia maturation in a commercially important fungal species. Future efforts will focus on the function of NDPK and the mechanism by which it regulates mycelia maturation.

## 1. Introduction

*Pleurotus tuoliensis*, also known as Bailinggu, is one of the most commercially important species of edible mushrooms [1]. *P. tuoliensis* was initially found in Xinjiang province [2] and is now widely commercially cultivated in China, Japan, and Korea, due to its desirable nutritional and medicinal value [3]. 

In recent years, *P. tuoliensis* yield decreased year by year. In 2017, only 70,000 tons were produced, which represents a 77% decrease from 2012 (http://hz.cefa.org.cn/2019/03/18/10498.html). This decrease may be due to this fungus’ biological characteristics, such as long cultivation period, a high proportion of deformed fruiting bodies, poor uniformity of fruiting bodies, and strain degeneration [4]. Together, these factors present challenges for mushroom farmers and thus lead to a decline in cultivation enthusiasm. Physiological maturation of *P. tuoliensis* mycelia is essential for its fruiting. This maturation takes 30–60 days and is responsible for the above-mentioned long cultivation period. In this physiological maturation stage, mycelia accumulate and metabolize nutrients [5]. Cold stimulation of physiologically mature mycelia can result in improved consistency in budding of primordia in culture bottles and ensures a high degree of fruiting body uniformity [6]. Our previous study demonstrated that N-carbamoyl-L-aspartate (CA-asp) can be used as an indicator of physiological maturation of mycelia. A mycelial CA-asp content of 0.9–1.2 g/L is thought to indicate physiological maturation and induces primordia formation and fruiting body growth [7]. The identification of such an indicator makes *P. tuoliensis* cultivation more tractable and aids efforts to improve *P. tuoliensis* quality and yield. However, the molecular mechanisms underlying physiological maturation of *P. tuoliensis* mycelia remain undefined. 

RNA-seq is a rapid and cost-effective technology for discovery of genes expressed in a given growth stage, especially in non-model species for which reference genome sequences are not available [8]. Many transcriptome studies have been performed to explore growth and development of mushroom species, including *Pleurotus eryngii* [9], *Cordyceps militaris* [10], and *Ganoderma lucidum* [11]. This high-throughput deep sequencing approach has also been successfully applied to investigate the mechanisms underlying response to cold stimulation in *P. tuoliensis* mycelia [6] as well as the identification of genes associated with *P. tuoliensis* fruiting body formation [12].

To our knowledge, no transcriptome analyses have been performed to investigate the transcriptional changes that occur during physiological maturation of *P. tuoliensis* mycelia. To identify genes and pathways that are differentially expressed during physiological mycelium maturation and explore molecular mechanisms underlying this maturation, a comparative transcriptome study on immature and mature *P. tuoliensis* mycelia was performed in this study. 

## 2. Materials and Methods

### 2.1. Collection of Pleurotus Tuoliensis Mycelia 

*P. tuoliensis* (ACCC 50869) was obtained from the Agricultural Culture Collection of China. Mycelia were grown at 25 °C in plastic bottles containing 820 g of cultivation substrate (40.43% cotton seed hull, 21.56% corncob, 25.16% wheat bran, 9.88% corn flour, 0.99% calcium carbonate, and 1.98% lime, and 65% water content, pH 8.5–8.8) in the dark for 37 days, after which the substrate was fully covered by mycelia. We randomly selected three cultivation bottles and took the mycelia from cultivation substrate into three corresponding tubes and froze them at −80 °C until RNA extraction (control samples, A). The remaining plastic bottles were maintained at a temperature of 17 °C in the dark to induce physiological maturation of mycelia. After 20 days of induction, CA-asp content in mycelia were determined every two days. After CA-asp content in mycelia reached 0.9 g/L, mycelia reached physiological maturation [7]. We then randomly selected three cultivation bottles and transferred the mycelia to three corresponding tubes. All mycelia samples were stored at −80 °C until RNA extraction (case samples, B). The remaining plastic bottles were transferred to a mushroom house for fruiting management. 

### 2.2. Library Preparation and RNA-Seq

Mycelia total RNA was extracted using TRIzol reagent (Life Technologies, New York, NY, USA) according to the manufacturer’s protocol. The concentration, quality, and integrity of the extracted RNA samples were determined by agarose gel electrophoresis and a NanoDrop spectrophotometer (Thermo Scientific, USA). Six cDNA libraries were constructed using the Illumina TruSeq Stranded mRNA LT Library Preparation Kit (Illumina, USA) and then paired-end sequenced on an Illumina HiSeq 2000 platform by Shanghai Personal Biotechnology Co. Ltd. (Shanghai, China). 

### 2.3. De Novo Transcriptome Assembly and Annotation

Raw reads were assessed for quality using FastQC (https://www.bibsonomy.org/bibtex/f230a919c34360709aa298734d63dca3) and have been deposited in the National Center for Biotechnology Information (NCBI) database under the the BioProject accession PRJNA551095. Low-quality reads, duplicate sequences and adapter sequences were removed to obtain high-quality clean reads using cutadapt (https://cutadapt.readthedocs.io/en/stable/). Clean paired-end reads were assembled into contigs based on the similarity between overlapping regions using Trinity [13]. The resulting contigs were clustered with TGICL-2.1 [14] with the parameters “-l40-v20”. The transcript isoforms with the longest sequence in each Trinity subcomponent were extracted as a representative sequence of the Trinity subcomponent, called Unigene. All unigenes were pooled to yield the final unigene library. 

Based on sequence homology, all assembled unigenes were compared to the NCBI non-redundant protein (Nr) and Swiss-Prot databases using the BLASTX program (*e*-value ≤ 1 × 10^−5^). The Blast2GO program [15] was used to obtain gene ontology (GO) annotations for the unigenes, and GO-term classification was conducted based on the Nr annotations. Pathway annotations were assigned using the BBH (bi-directional best hit) method of the Kyoto Encyclopedia of Genes and Genomes (KEGG) Automatic Annotation Server (KAAS) online tool [16]. Unigenes were also aligned to the Evolutionary Genealogy of Genes: Non-Supervised Orthologous Groups (eggNOG) database for further functional prediction and classification.

### 2.4. Identification and Functional Analysis of Differentially Expressed Genes (DEGs) 

To identify the differentially expressed genes, transcript abundance were first calculated by mapping clean reads to unigenes database using RSEM [17]. DEGs were then identified by R Bio-package DEseq2. [18]. The significance of gene expression differences was assessed using the *p*-value ≤ 0.05 and |log2 ratio| ≥ 1. These differentially expressed data have been deposited in the Gene Expression Omnibus (GEO) database at the NCBI archives (https://www.ncbi.nlm.nih.gov/geo) under accession GSE135839. 

The online analysis tool, the Database for Annotation, Visualization and Integrated Discovery (DAVID; https://david.ncifcrf.gov/) were used to analysis DEGs for GO term and KEGG pathyway enrichment. 

### 2.5. Reverse Transcription-Quantitative PCR (RT-qPCR) Analysis

We selected 12 DEGs identified by RNA-seq for validation by RT-qPCR. Total RNA from immature and mature mycelia was extracted using the same procedures as used for RNA-seq. RNA from each sample was reverse transcribed using TransScript One-Step gDNA Removal and cDNA Synthesis SuperMix (TransGen Biotech, Beijing, China). Primers for the 12 DEGs were designed in Primer 6.0 software (Premier, Ottawa, Canada) and are shown in Table 1. Reaction mixtures (20 µL) contained 10 µL of 2× SYBR Green Master Mix, 0.4 µL of 10 nM each primer, 2 µL of cDNA template, and 7.2 µL of ddH_2_O. The qPCR reactions were cycled on an ABI Prism 7500 Real-Time PCR System (Applied Biosystems, Foster City, CA, USA) with SYBR green fluorescent dye (TaKaRa, Dalian, China). Amplification conditions were: 95 °C for 3 min, followed by 40 cycles of 94 °C for 3 s, 60 °C for 32 s, and 72 °C for 30 s. Each reaction was performed in triplicate using the glyceraldehyde-phosphate dehydrogenase gene (*gapdh*) as an internal reference. Relative gene expression levels were calculated using the 2^−∆∆Ct^ method [19].

## 3. Results

### 3.1. Growth of P. Tuoliensis Mycelia

**Figure 1:**
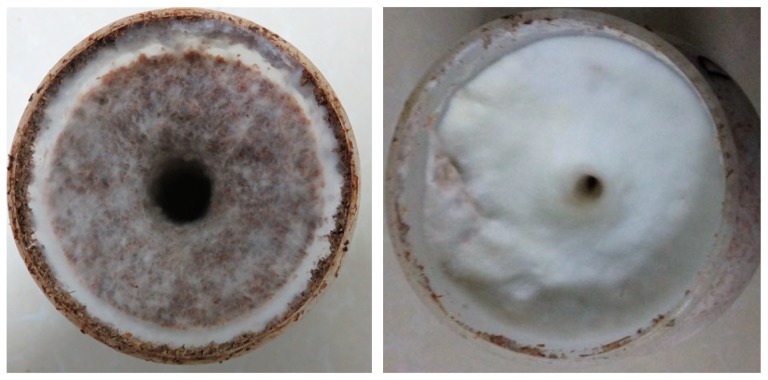
The morphological characteristics of immature mycelia (Left image) and mature mycelia (Right image).

The cultivation substrate was fully covered by white fungal mycelia after incubation at 25 °C for 37 days in the dark. These cultivation bottles were then maintained at a temperature of 17 °C in the dark to induce mycelia maturation. The sawdust media surface was gradually covered with plump and elastic hyphae (Figure 1). At the 35th day, CA-asp content in mycelia was determined to be 0.982 g/L, which means the physiological maturation of mycelia. Primordia were initiated within 7 days under low temperature and light stimulation conditions and within 15 days, developed reproductive fruiting bodies that could be harvested. To investigate the molecular basis of physiological maturation of mycelia, we collected immature mycelia and mature mycelia for RNA-seq analysis.

### 3.2. Sequencing and Gene Annotation

**Figure 2:**
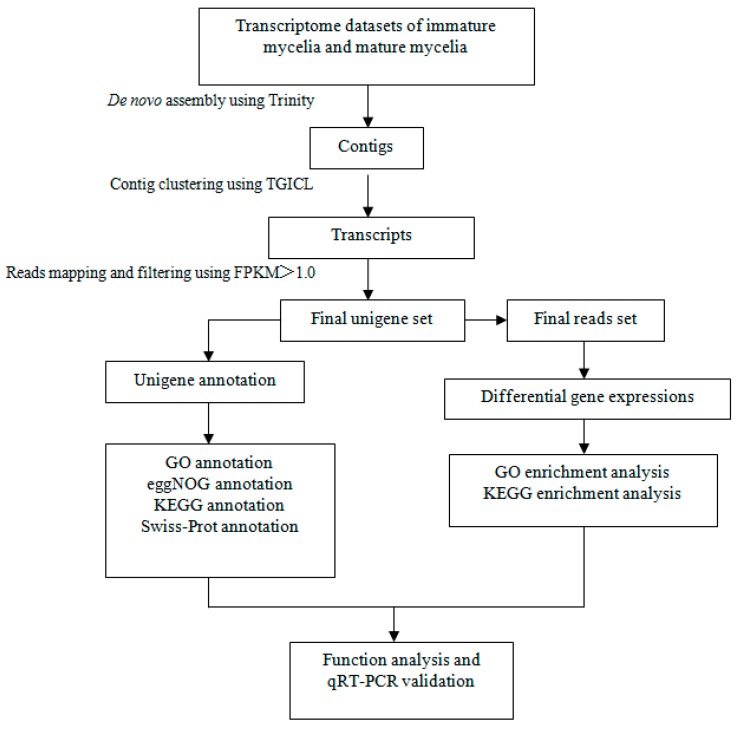
Analytical framework of the *P. tuoliensis* mycelia transcriptome. Raw reads were filtered and then de novo assembled into contigs by Trinity. Unigenes were obtained by clustering the contigs with TGICL. FPKM values were calculated for each of the unigenes, and only unigenes with FPKM > 1.0 were used for further analyses and annotation against the public databases.

We performed paired-end sequencing of six cDNA libraries (three biological replicates each for immature and mature mycelia). We obtained an average of 45,688,081 raw reads per library. After removing adapter sequences and low-quality reads, clean reads represented approximately 99.53% of the raw reads. Clean reads were combined into a single dataset for de novo assembly. A total of 60,330 transcripts, including sequence isoforms, were obtained. Transcripts displayed a mean length of 1897 bp. To reduce redundancy, only the longest sequence isoform within each subcomponent was selected. In total, we obtained 17,030 unigenes representing unique transcripts for further analysis (Figure 2). 

This unigene dataset displayed a total size of 21,120,061 bp, an average length of 1240 bp, and an N50 of 1891 and served as the mRNA transcriptome for mycelia (Table 2). 

In total, 14,300 and 7115 unigenes were mapped using the eggNOG (Table S1) and Swiss-Prot (Table S2) databases, respectively. In addition, 13,611 (79.92%) unigenes matched known proteins in NR (NCBI non-redundant protein sequences) database (Table S3), and 95% of these were homologous with proteins from *P. ostreatus*, a species evolutionarily related to *P. tuoliensis* (Table S4). Additionally, 8,804 (51.70%) unigenes assigned to more than one GO term were classified into 67 functional groups from the Gene Ontology database (Table S5). Using KAAS (KEGG Automatic Annotation Server) with *P. ostreatus* as the reference, a total of 2,942 unigenes were mapped to 35 level-2 pathways (Table S6). Furthermore, a total of 2603 unigenes were annotated in all of these databases.

### 3.3. Identification and Functional Enrichment Analysis of Differentially Expressed Genes (DEGs) 

**Figure 3:**
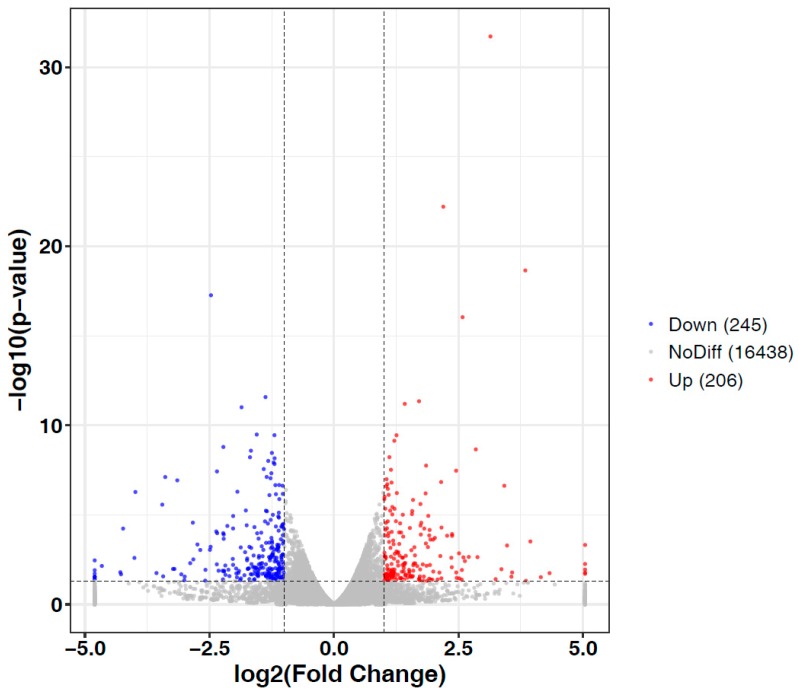
The volcano plots showing expression level of each unigene. Limits defined by *p*-value ≤ 0.05 and |log2 ratio| ≥ 1. Red points represent upregulated genes; blue points represent downregulated genes; gray area represents insignificant gene.

To identify significantly differentially expressed genes (DEGs), we compared the normalized read counts (FPKM values) of the immature and mature mycelia samples. This comparative analysis revealed 451 significantly DEGs (*p*-value ≤ 0.05, |log_2_ ratio| ≥ 1), which included 206 upregulated and 245 downregulated genes (Figure 3). The analyzed data of differential expression analysis for each unigene are shown in Table S7.

**Figure 4:**
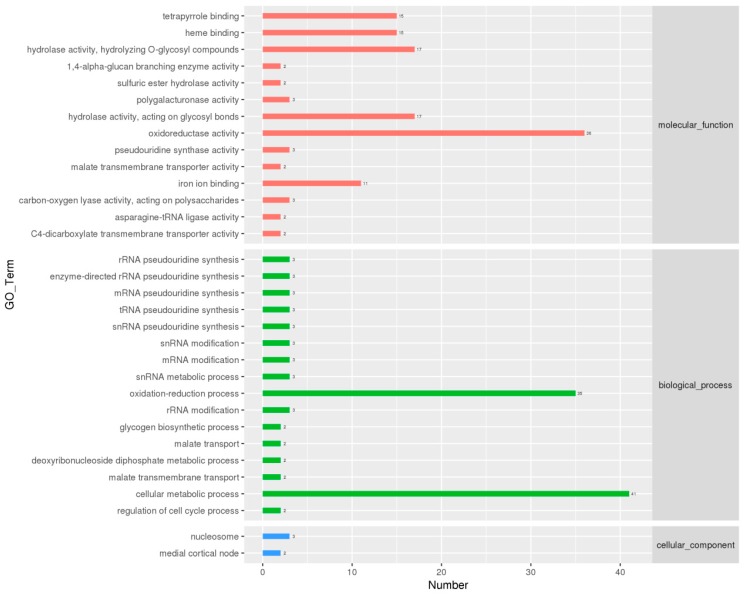
GO functional classification of differentially expressed genes. The green bars represent biological process; yellow bars represent cellular component; blue bars represent molecular function.

We next performed GO analysis on the DEGs to gain insight into their biological functions. A total of 197 DEGs were enriched. Enrichment result of all DEGs is shown in Table S8. Figure 4 shows the top 32 enriched GO terms assigned to our DEGs. The cellular metabolic process (GO:0044237) annotation was enriched with most DEGs (False discovery rate (FDR) > 0.05). Oxidation-reduction process (GO:0055114) and oxidoreductase activity (GO:0016491) were also enriched (FDR > 0.05), indicating that oxidation-reduction processes may be highly active in mature mycelia. Other enriched GO terms were mainly involved in the synthesis and processing of genetic information, including rRNA modification (GO:0000154) (FDR > 0.05), mRNA pseudouridine synthesis (GO:1990481) (FDR < 0.05), snRNA metabolic process (GO:0016073) (FDR > 0.05), and deoxyribonucleoside diphosphate metabolic process (GO:0009186) (FDR > 0.05). Within the molecular function category, terms associated with carbohydrate metabolism, such as hydrolase activity acting on O-glycosyl compounds (GO:0004553) (FDR > 0.05) and hydrolase activity acting on glycosyl bonds (GO:0016798) (FDR > 0.05), were enriched. In general, GO terms associated with genetic information synthesis and processing were largely enriched. 

**Figure 5:**
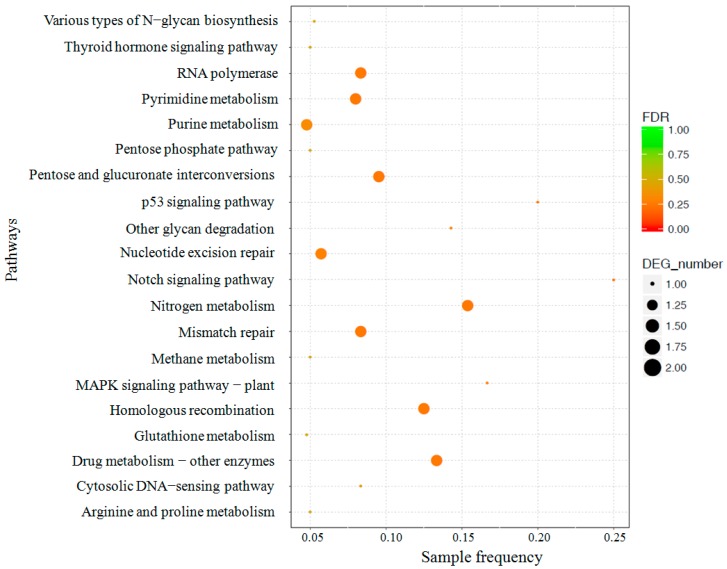
The top 20 enriched KEGG pathways of differentially expressed genes between immature mycelia and mature mycelia. ‘Sample frequency’ means the ratio of the number of differentially expressed genes in a pathway to the number of all annotated genes in this pathway in the figure legend. DEG number indicates the number of differential genes annotated in the pathway, indicated by a dot, the larger the dot, the more differential genes are enriched. The color gradient indicates the corrected *p*-value by Benjamini–Hochberg FDR method. Only pathways with FDR < 1 were shown.

To better understand the functions and interactions of our DEGs, all DEGs underwent a pathway-based analysis by mapping against the KEGG database. Pathway enrichment analysis demonstrated that only 35 pathways were enriched, which included 45 DEGs (Table S9). We found that these pathways were mainly associated with nucleotide metabolism, DNA replication and repair, and energy metabolism, such as pyrimidine metabolism (ko00240), nucleotide excision repair (ko03420), pyruvate metabolism (ko00620), and the pentose phosphate pathway (ko00030). The results of our KEGG analysis corresponded with those obtained by our GO enrichment analysis. The 20 enriched pathways with FDR < 1 are shown in Figure 5.

In addition, the total annotation results of Unigenes and DEGs are listed in Table S10.

### 3.4. Identification of DEGs Related to Physiological Maturation of Mycelia

**Figure 6:**
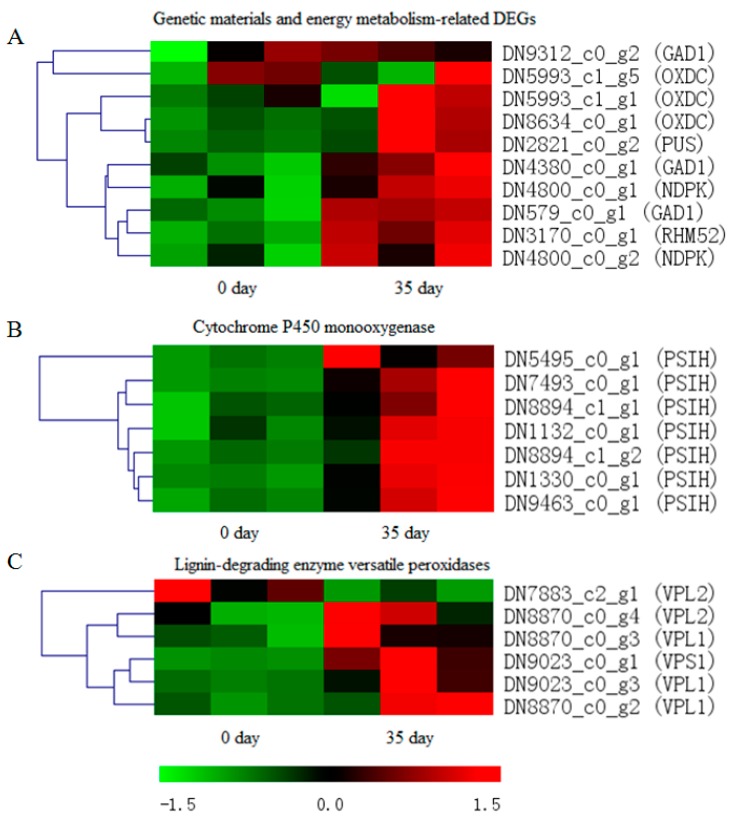
Genes involved in the regulation of mycelia physiological maturation in *P. tuoliensis*. (**A**) DEGs associated with genetic material and energy metabolism. (**B**) DEGs associated with metabolic processes and stress responses. (**C**) Lignin-degrading enzymes versatile peroxidases. The right side of the heatmap indicates the gene ID in our transcriptomic data for *P. tuoliensis* and the homologous gene name. The gene expression values (FPKMs) were transformed to *z*-score values.

To identify genes related to physiological maturation of mycelia, we analyzed significantly upregulated DEGs in mature mycelia. Overall, these upregulated DEGs are mainly involved in the synthesis of genetic materials and energy metabolism, and include unigenes encoding glutamate decarboxylase Gad1 (DN579_c0_g1), oxalate decarboxylase OxdC (DN8634_c0_g1), pseudouridylate synthase Pus (DN2821_c0_g2), DNA repair and recombination protein RHM52 (DN3170_c0_g1), and nucleoside diphosphate kinase Ndpk (DN4800_c0_g2), demonstrating that this complex developmental program requires not only fast mycelial vegetative growth, but also more energy to accumulate nutrients and thus nurture developing fruit bodies during the reproductive stage. Versatile peroxidases (Vps) are key lignin-degrading enzymes, as they possess both manganese and lignin peroxidase activities. We observed upregulation of Vp-encoding genes (DN9023_c0_g1, DN9023_c0_g3, and DN8870_c0_g2) in mature mycelia, consistent with nutrient accumulation. Another important class of DEGs identified in our mature mycelia were those encoding post-modification enzymes, such as cytochrome P450 monooxygenase and methyltransferases, which are important for the synthesis of secondary metabolites. The expression of genes encoding cytochrome P450s monooxygenase Psih (DN1132_c0_g1, DN1330_c0_g1, DN5495_c0_g1, DN7493_c0_g1, DN8894_c1_g1, DN8894_c1_g2, and DN9463_c0_g1) and a methyltransferase (DN4644_c0_g2) were found to be significantly upregulated in mature mycelia. Finally, upregulated fruiting body protein SC3 (DN5369_c0_g1) was enriched in mature mycelia, probably indicating the initiation and formation of fruiting bodies (Figure 6). 

### 3.5. Distinct Distribution of Carbohydrate-Active Enzymes during Mycelial Maturation

**Figure 7:**
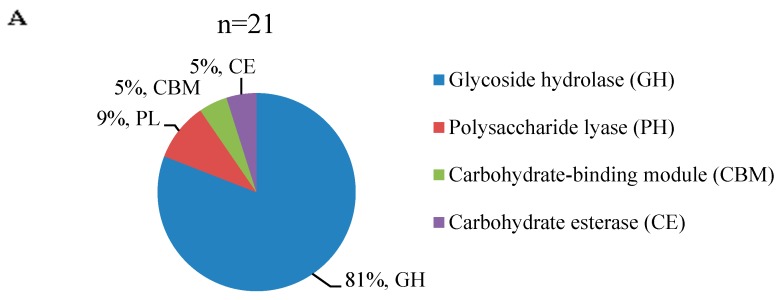

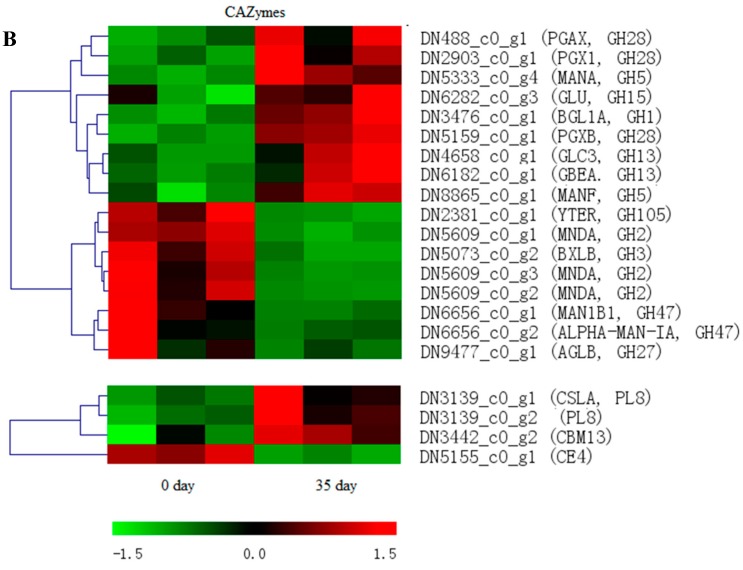
CAZymes analysis involved in mycelia physiological maturation. (**A**) the distribution of CAZymes identified. The corresponding genes were searched using the carbohydrate-active enzyme database (CAZy) and were classified into primary domains such as glycoside hydrolase (GH), carbohydrate-binding module (CBM), carbohydrate esterase (CE), glycosyl transferase (GT), and polysaccharide lyase (PL). (**B**) The expression heatmap of these genes.

In this study, we also searched our DEGs for families related to carbohydrate-active enzymes (CAZymes) using the Carbohydrate Active Enzymes (CAZy) database [20]. BLASTX indicated that a total of 21 DEGs from the mycelia transcriptome were CAZymes. These upregulated CAZymes could be classified into superfamilies, such as glycoside hydrolases (GHs), polysaccharide lyases (PLs), carbohydrate-binding modules (CBMs), and carbohydrate esterase (CE) superfamily (Figure 7A), which likely indicates a functional change in plant cell wall polysaccharide degradation in cultivation substrate. The differences in expression of these CAZymes between immature and mature mycelia are summarized in Figure 7B. The genes encoding exopolygalacturonase (DN2903_c0_g1, GH28), β-glucosidase (DN3476_c0_g1, GH1), 1,4-α-glucan-branching enzyme (DN4658_c0_g1, GH13), glucoamylase (GH15), and mannan endo-1,4-β-mannosidase (DN5333_c0_g4, GH5) were upregulated in mature mycelia, and β-mannosidase (GH2) and α-galactosidase (GH27) were downregulated.

### 3.6. Validation of DEGs by RT-qPCR

**Figure 8:**
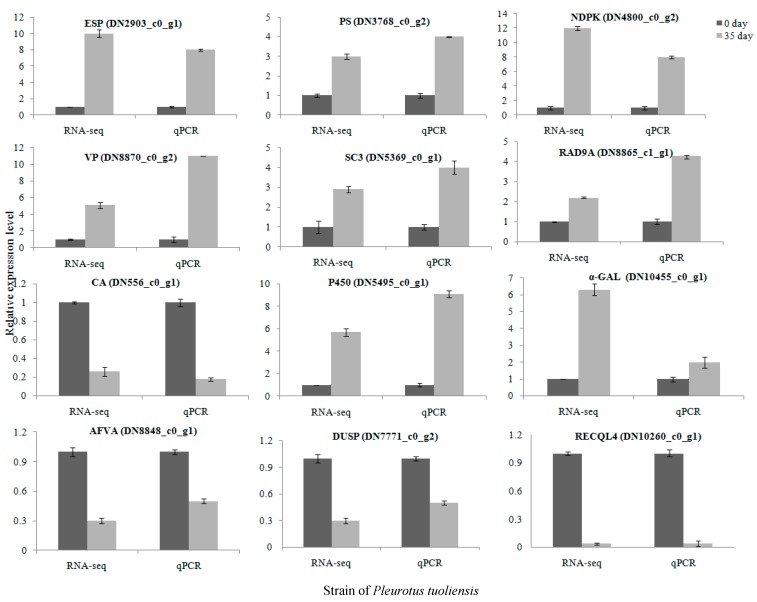
Expression of 12 selected genes as determined by RT-qPCR in comparison to the RNA-seq results. *Gapdh* expression was used as the internal control. The RT-qPCR values for each gene are means ± SD of three biological replica.

Reverse transcription-quantitative PCR (RT-qPCR) was performed to verify the expression of 12 DEGs, including exopolygalacturonase ESP (DN2903_c0_g1), pseudouridine synthase PS (DN3768_c0_g2), nucleoside diphosphate kinase NDPK (DN4800_c0_g2), versatile peroxidase VP (DN8870_c0_g2), fruiting body protein SC3 (DN5369_c0_g1), cytochrome P450 monooxygenase (DN5495_c0_g1), cell cycle checkpoint control protein RAD9A (DN8865_c1_g1), α-galactosidase α-GAL (DN10455_c0_g1), carbonic anhydrase CA (DN556_c0_g1), NADPH dehydrogenase afvA (AFVA), dual specificity protein phosphatase (DUSP), and ATP-dependent DNA helicase Q4 (RECQL4). All of these DEGs showed similar expression patterns in RT-qPCR analysis as observed from RNA-seq data (Figure 8).

## 4. Discussion

The physiological maturation stage of mycelia, which lasts approximately 30–60 days, is critical for the growth and development of the *P. tuoliensis* fruiting body. Only mature mycelia are subjected to low temperature and light stimulation, primordia and fruiting body can be finely developed. Transcriptomic studies on mature mycelia to cold stimulation and fruiting body formation for *P. tuoliensis* have been performed by Fu et al. (2016, 2017) to reveal physiological metabolic properties underlying primordia formation and fruiting body development [6,12]. Transcriptomic analysis on cold stimulation of *P. tuoliensis* mycelia indicated that functional groups of differentially expressed unigenes associated with cell wall and membrane stabilization, soluble sugars, and protein biosynthesis and metabolism pathways, and calcium signaling and mitogen-activated protein kinases (MAPK) pathways play a vital role in Bailinggu’s response to cold stimulation. While transcriptomic analysis on fruiting body development identified the stage-specific and differentially expressed unigenes (DEGs) involved in morphogenesis, primary carbohydrate metabolism, cold stimulation, and blue-light response, predicting that these unigenes might help *P. tuoliensis* adapt to genetic and environmental factors that influence fructification. However, no transcriptome analyses have been performed to study the molecular mechanisms underlying physiological maturation of mycelia. In this study, we utilized a comparative transcriptomic approach to explore differential gene expression and molecular processes during the physiological maturation of mycelia. 

Our comparative transcriptome analysis identified 451 differentially expressed genes (DEGs) between immature and mature mycelia. Although the number of DEGs is small considering ~17000 unigenes, this result is reasonable. Even if *P. tuoliensis* mycelia have undergone a physiological maturation process, their morphology does not display a huge change relative to the process of primordia formation or fruiting body development. As shown in GO and KEGG enrichment results of DEGs, DEGs were mainly enriched for nucleotide synthesis and energy metabolism functions. Which are consistent with the fast growth and nutrient accumulation that are characteristic of mycelium maturation [21]. Metabolome analysis of physiological maturation of *P. tuoliensis* mycelia also revealed similar metabolism phenomenon. Metabolites enriched on pyrimidine synthesis were significantly abundant in mature *P. tuoliensis* mycelia, such as CMP and cytosine, which are indicative of the high demand for rapid nucleotide synthesis during physiological maturation [7].

Among our DEGs, the upregulation expression of NDPK is interesting. Many studies have demonstrated that NDPK regulates various biological processes and signal transduction pathways in addition to playing a fundamental role in maintaining the cellular balance of NDP and NTP [22,23]. In *Neurospora crassa*, Ndpk is associated with hyphal development and photomorphogenesis [24]. Tang et al. (2016) reported that Ndpk was significantly upregulated in mature *Lentinus edodes* mycelia, concluding that this enzyme may plays an important role in light-induced mycelial brown-film formation, an indicator of physiological mycelial maturation in *L. edodes* [21]. Thus, upregulation of NDPK in mature *P. tuoliensis* mycelia is likely associated with physiological mycelial maturation. The specific function of Ndpk in this process is worthy of further study.

Growth and development of *P. tuoliensis* relies on nutrients produced by decomposition of the cultivation substrate. CAZymes are involved in the hydrolysis of plant cell wall polysaccharides and play an important role in substrate degradation processes [25]. We identified a total of 21 CAZymes, most of which belong to glycoside hydrolase (GH) families, which play important roles in lignocellulose decomposition [26]. The GH families GH13 (2 genes), GH15 (1 gene), GH28 (3 genes), GH5 (2 genes), and GH1 (1 gene) were upregulated in our mature mycelia. GH5 is one of the largest GH families, whose members act on a wide range of substrates, including β-1,3-glucans, β-1,4-glucans in cellulose, and β-1,4-mannans in hemicelluloses [27]. GH13 is a family of starch-debranching enzymes that hydrolyze the α-1,6-glucosidic bond in starch, and GH28 members play critical roles in pectin degradation [28]. The function of GH15 is not known. Lignin degradation involves three types of degrading enzymes: manganese peroxidases (MnPs) [29], versatile peroxidases (VPs) [30], and laccases [31]. We identified three upregulated VP-encoding genes in mature mycelia. These upregulated genes are probably related with lignin degradation. 

Functional enrichment analyses of our DEGs also demonstrated that oxidation-reduction activities and pathways are very active during the physiological maturation of mycelia. Cytochrome P450 monooxygenases psiH, which catalyze the biooxidation of various substrates through activation of molecular oxygen and play important roles in metabolic processes and stress responses [32], displayed increased expression in mature mycelia. Similar expression patterns for these genes were observed by qPCR analysis. Identification of upregulated PSIH demonstrated that mycelium maturation requires increased oxygen and produces excess water, which often leads to submergence and hypoxic stress. 

We also observed upregulated fruiting body protein SC3 in mature mycelia, which was reported to be involved in fruiting body development of edible mushroom [33,34]. The high abundance of this protein probably indicates the initiation and formation of fruiting bodies. Fu et al. (2017) found that unigenes encoding fruiting body hydrophobin 1 were significantly upregulated in primordia and fruiting body for *P. tuoliensis*. The unigene encoding hydrophobin 2 was upregulated in primordia while downregulated in the fruiting bodies. However, they did not find unigenes that specifically expressed during the initiation of fruiting [12].

In summary, our comparative transcriptomic analysis of immature and mature mycelia revealed gene expression differences indicative of changing metabolism during physiological mycelium maturation and identified a potential functional group of genes responsible for regulating the physiological maturation in *P. tuoliensis* mycelia. GO and KEGG enrichment analysis demonstrated that metabolic pathways related to genetic information and energy metabolism are extremely active during the physiological maturation of mycelia. Genes related to critical oxidation-reduction processes are upregulated in mature mycelia. CAZymes involved in the hydrolysis of plant cell wall polysaccharides were differentially expressed in mature mycelia compared with immature mycelia and the differential expression of these genes may relate with substrate degradation utilization. In addition, we predict that NDPK may be an important enzyme for participating in mycelia physiological maturation. Our data enables an improved understanding of physiological maturation in *P. tuoliensis* mycelia, which lay an important foundation for revealing mycelia physiological maturation properties in edible mushrooms.

## Figures and Tables

**Table 1:** Primers for qRT-PCR of 12 DEGs.

Gene ID	Annotation	Primer
DN2903_c0_g1	Exopolygalacturonase (EPS)	F:GTGAGTTGGCTTGCTTCT R:CGATTCCTATCTGTCTTGGT
DN3768_c0_g2	Pseudouridine synthase (PS)	F:TCGTCTACCTCCATATTGTC R: CTCTCAGCACCATACCTAG
DN4800_c0_g2	Nucleoside diphosphate kinase (NDPK)	F: GCTGTTGGACGCAATATC R:CACACTTCCTAAGACTTCAC
DN8870_c0_g2	Versatile peroxidase (VP)	F:GCAATGATGAAGACACAGAC R: GCAACTACGCAGCCTAAT
DN5369_c0_g1	Fruiting body protein SC3 (SC3)	F: CTTCATCGCCTATCTTCACT R: CGCACCAATAACAGTAACG
DN5495_c0_g1	Cytochrome P450 monooxygenase (P450)	F: TCCTTCGTATTCGTCTTCAG R: CGTCGCTACCTTACATCAA
DN8865_c1_g1	Cell cycle checkpoint control protein RAD9A (RAD9A)	F: GCATCACCGTCCATCATT R: CTTCCGCAATCTCGTCTT
DN556_c0_g1	Carbonic anhydrase (CA)	F:CGTCTCATCTCTGTCACTAAT R: CCGAGGTGTTCAGGAATC
DN10455_c0_g1	α-galactosidase (α-GAL)	F: CAGAATACGACCGCATGG R: AAGCACCACCAACCAATG
DN8848_c0_g1	NADPH dehydrogenase afvA (AFVA)	F: CCTTCCTATCGTCTTCTCTG R: CCACTTCAACTGCCTCTC
DN7771_c0_g2	Dual specificity protein phosphatase (DUSP)	F: AGGAACGACTGAACTGAG R: GGTATGGTGGAGGTGAAG
DN10260_c0_g1	ATP-dependent DNA helicase Q4 (RECQL4)	F: GTCTGTTGCTGCTTGTCT R: CTCTATCCATTGCTTGATGTC

**Table 2:** Assembly and annotation statistics of *P. tuoliensis* mycelia transcriptome.

Samples	PE01	PE02	PE03	PE351	PE352	PE353
Raw reads	41,410,374	46,175,662	43,409,086	45,505,956	48,825,340	48,802,066
Clean reads	41,222,978	45,964,590	43,208,980	45,306,508	48,616,554	48,565,980
Percentage of clean reads (%) ^a^	99.54	99.54	99.53	99.56	99.57	99.51
Assembled statistics						
Number of assembled transcripts			60,330			
Mean length of assembled transcripts (bp)			1897			
Number of assembled unigene			17,030			
Total assembled unigene size (bp)			21,120,061			
Mean length of assembled unigene (bp)			1240			
N50 (bp) ^b^			1891			
The number of N50 transcripts			3412			
Annotation						
NR			13,611			
GO			8804			
KEGG			2942			

^a^ Percentage of the number of clean reads in the number of total raw reads. ^b^ All sequences are arranged from long to short sequence, and the sequence lengths are sequentially added in this order, When the added length reaches 50% of the total length of the sequence, the length of the last sequence is recorded as N50 (bp).

## Supplementary Materials

The following are available online at https://www.mdpi.com/2073-4425/10/9/703/s1, Table S1: eggNOG annotation statistics of unigenes; Table S2: unigenes annotation information in Swiss-Prot databases; Table S3: unigenes matched known proteins in NR database; Table S4: Nr annotation statistics of unigenes; Table S5: GO enrichment terms of unigenes; Table S6: KEGG pathways enriched with unigenes; Table S7: the total data of differential expression analysis for each unigene; Table S8: GO enrichment terms of differential expressed genes; Table S9: KEGG enrichment pathways of differential expressed genes; Table S10: the total annotation of unigenes and differential expressed genes.

## References

[1] Zhao M.R., Zhang J.X., Chen Q., Wu X.L., Gao W., Deng W.Q., Huang C.Y. (2016). The famous cultivated mushroom Bailinggu is a separate species of the *Pleurotus eryngii* species complex. Sci. Rep..

[2] Kawai G., Babasaki K., Neda H. (2008). Taxonomic position of a Chinese Pleurotus “Bai-Ling-Gu”: It belongs to *Pleurotus eryngii* (DC.: Fr.) Quél. and evolved independently in China. Mycoscience.

[3] Wang C.L., Cui H.Y., Wang Y.R., Wang Z.F., Li Z.J., Chen M.H., Li F.J. (2014). Bidirectional immunomodulatory activities of polysaccharides purified from *Pleurotus nebrodensis*. Inflammation.

[4] Wang S.X., Zhao S., Huang Z.X., Yin L.M., Hu J., Li J.H., Liu Y., Rong C.B. (2018). Development of a highly productive strain of *Pleurotus tuoliensis* for commercial cultivation by crossbreeding. Sci. Hortic..

[5] Gou Y.P., He Y.W., Mao Z.Y. (2003). Effect of after-riping time on the fruitng and yield of *Pleurotus tuoliensis*. Mushroom.

[6] Fu Y.P., Liang Y., Dai Y.T., Yang C.T., Duan M.Z., Zhang Z., Hu S.N., Zhang Z.W., Li Y. (2016). *De novo* sequencing and transcriptome analysis of *Pleurotus eryngii* subsp. *tuoliensis* (Bailinggu) mycelia in response to cold stimulation. Molecules.

[7] Du F., Zou Y.J., Hu Q.X., Jing Y.G., Yang X.H. (2019). Metabolic profiling of *Pleurotus tuoliensis* during mycelium physiological maturation and exploration on a potential indicator of mycelial maturation. Front. Microbiol..

[8] Wong M.M., Cannon C.H., Wickneswari R. (2011). Identification of lignin genes and regulatory sequences involved in secondary cell wall formation in *Acacia auriculiformis* and *Acacia mangium* via *de novo* transcriptome sequencing. BMC Genom..

[9] Xie C.L., Gong W.B., Zhu Z.H., Yan L., Hu Z.X., Peng Y.D. (2018). Comparative transcriptomics of *Pleurotus eryngii* reveals blue-light regulation of carbohydrate-active enzymes (CAZymes) expression at primordium differentiated into fruiting body stage. Genomics.

[10] Yin J., Xin X.D., Weng Y.J., Gui Z.Z. (2017). Transcriptome-wide analysis reveals the progress of *Cordyceps militaris* subculture degeneration. PLoS ONE.

[11] Yu G.J., Wang M., Huang J., Yin Y.L., Chen Y.J., Jiang S., Jin Y.X., Lan X.Q., Wong B.H.C., Liang Y. (2012). Deep insight into the *Ganoderma lucidum* by comprehensive analysis of its transcriptome. PLoS ONE.

[12] Fu Y.P., Dai Y.T., Yang C.T., Wei P., Song B., Yang Y., Sun L., Zhang Z.W., Li Y. (2017). Comparative transcriptome analysis identified candidate genes related to Bailinggu mushroom formation and genetic markers for genetic analyses and breeding. Sci. Rep..

[13] Grabherr M.G., Haas B.J., Moran Y., Levin J.Z., Thompson D.A., Ido A., Xian A., Lin F., Raktima R., Qiandong Z. (2011). Full-length transcriptome assembly from RNA-Seq data without a reference genome. Nat. Biotechnol..

[14] Pertea G., Huang X.Q., Liang F., Antonescu V., Sultana R., Karamycheva S., Lee Y.D., White J., Cheung F., Parvizi B. (2003). TIGR gene indices clustering tools (TGICL): A software system for fast clustering of large EST datasets. Bioinformatics.

[15] Stefan G.T., Juan Miguel G.G., Javier T., Williams T.D., Nagaraj S.H., María José N., Montserrat R., Manuel T., Joaquín D., Ana C. (2008). High-throughput functional annotation and data mining with the Blast2GO suite. Nucleic Acids Res..

[16] Yuki M., Masumi I., Shujiro O., Yoshizawa A.C., Minoru K. (2007). KAAS: An automatic genome annotation and pathway reconstruction server. Nucleic Acids Res..

[17] Li B., Dewey C.N. (2011). RSEM: Accurate transcript quantification from RNA-seq data with or without a reference genome. BMC Bioinform..

[18] Love M.I., Huber W., Anders S. (2014). Moderated estimation of fold change and, dispersion for RNA-seq data with DESeq. 2. Genome Biol..

[19] Livak K.J., Schmittgen T.D. (2001). Analysis of relative gene expression data using real-time quantitative PCR and the 2-DDCt method. Methods.

[20] Cantarel B.L., Coutinho P.M., Corinne R., Thomas B., Vincent L., Bernard H. (2009). The Carbohydrate-Active EnZymes database (CAZy): An expert resource for Glycogenomics. Nucleic Acids Res..

[21] Tang L.H., Tan Q., Bao D.P., Zhang X.H., Jian H.H., Li Y., Yang R.H., Wang Y. (2016). Comparative proteomic analysis of light-induced mycelial brown film formation in *Lentinula edodes*. BioMed Res. Int..

[22] Hetmann A., Kowalczyk S. (2009). Nucleoside diphosphate kinase isoforms regulated by phytochrome A isolated from oat coleoptiles. Acta Biochim. Pol..

[23] Hasunuma K., Yabe N., Yoshida Y., Ogura Y., Hamada T. (2003). Putative functions of nucleoside diphosphate kinase in plants and fungi. J. Bioenerg. Biomembr..

[24] Lee B., Yoshida Y., Hasunuma K. (2009). Nucleoside diphosphate kinase-1 regulates hyphal development via the transcriptional regulation of catalase in *Neurospora crassa*. FEBS Lett..

[25] André I., Potocki-Véronèse G., Barbe S., Moulis C., Remaud-Siméon M. (2014). CAZyme discovery and design for sweet dreams. Curr. Opin. Chem. Biol..

[26] Sathya T.A., Khan M. (2014). Diversity of glycosyl hydrolase enzymes from metagenome and their application in food industry. J. Food Sci..

[27] Couturier M., Roussel A., Rosengren A., Leone P., Stalbrand H., Berrin J.G. (2013). Structural and biochemical analyses of glycoside hydrolase families 5 and 26 β-(1,4)-mannanases from *Podospora anserina* reveal differences upon manno-oligosaccharide catalysis. J. Biol. Chem..

[28] Sathya T.A., Jacob A.M., Khan M. (2014). Cloning and molecular modelling of pectin degrading glycosyl hydrolase of family 28 from soil metagenomic library. Mol. Biol. Rep..

[29] Giardina P., Palmieri G., Fontanella B., Rivieccio V., Sannia G. (2000). Manganese peroxidase isoenzymes produced by *Pleurotus ostreatus* grown on wood sawdust. Arch. Biochem. Biophys..

[30] Camarero S., Sarkar S., Ruiz-Duenas F.J., Martinez M.J., Martinez A.T. (1999). Description of a versatile peroxidase involved in the natural degradation of lignin that has both manganese peroxidase and lignin peroxidase substrate interaction sites. J. Biol. Chem..

[31] Rico A., Rencoret J., Del Río J.C., Martínez A.T., Gutiérrez A. (2014). Pretreatment with laccase and a phenolic mediator degrades lignin and enhances saccharification of Eucalyptus feedstock. Biotechnol. Biofuels.

[32] Werck-Reichhart D., Feyereisen R. (2000). Cytochromes P450: A success story. Genome Biol..

[33] Joh J.H., Lee S.H., Lee J.S., Kim K.H., Jeong S.J., Youn W.H., Kim N.K., Son E.S., Cho Y.S., Yoo Y.B. (2007). Isolation of genes expressed during the developmental stages of the oyster mushroom, Pleurotus ostreatus, using expressed sequence tags. FEMS Microbiol. Lett..

[34] Lee S.H., Jolr J.H., Lee J.S., Lim J.H., Kim K.Y., Yoo Y.B., Lee C.S., Kim B.G. (2009). Isolation of genes specifically expressed in different developmental stages of *Pleurotus ostreatus* using macroarray analysis. Mycobiology.

